# Knowledge, Perception and Barriers to Cancer Pain Management among Doctors in Malaysia

**DOI:** 10.21315/mjms2023.30.3.17

**Published:** 2023-06-27

**Authors:** Ting-Yi Kweh, Chian-Hui Yeoh, Huan-Keat Chan, Fazlina Ahmad

**Affiliations:** 1 Palliative Care Unit, Department of Medicine, Hospital Sultanah Bahiyah, Kedah, Malaysia; 2 Clinical Research Centre, Hospital Sultanah Bahiyah, Kedah, Malaysia

**Keywords:** cancer pain management, morphine, knowledge, attitude, perception, barrier

## Abstract

**Background:**

Pain remains common in people living with advanced cancer and is often inadequately managed. This study was designed to assess knowledge, perceptions and barriers to morphine use in cancer pain management among doctors in Malaysia.

**Methods:**

Doctors from multiple disciplines in a general hospital were invited to complete a 39-item self-reported questionnaire between November 2020 and December 2020. Each question was based on a 5-point Likert scale (1 = strongly disagree; 5 = strongly agree). ‘Agree’ and ‘strongly agree’ were considered correct or positive responses, except for nine questions worded in the opposite direction. Associations between variables were confirmed using Pearson’s chi-squared and Fisher’s exact tests.

**Results:**

Most respondents were house officers (206/321; 64.2%) with less than two years of service, followed by medical officers (68/321; 21.2%) and specialists (47/321; 14.6%). Only 7.2% of the respondents had received formal palliative care training before the study. Of the respondents, 73.5% were aware of the World Health Organization (WHO) analgaesic ladder, 60.7% were correct on oral morphine as the first line for moderate to severe cancer pain treatment and 91.9% knew the need to add rescue morphine for breakthrough pain. Additionally, 34.0% (*P* < 0.001) perceived morphine use caused addiction, 57.9% (*n* = 186) expressed fear of respiratory depression and 18.3% of medical officers and specialists perceived limited access and a maximum dose to prescribe. There was a significant difference in knowledge and perception between junior doctors and senior clinicians. The majority strongly agreed and agreed that there were inadequate training opportunities in cancer pain management.

**Conclusion:**

Inconsistent knowledge and negative perceptions of cancer pain management among doctors were demonstrated in this study.

## Introduction

Pain is highly prevalent among patients with cancer, especially in the metastatic and advanced stages (1). Patients consistently cited uncontrolled pain as one of the sources of fear in the end-of-life phase (2, 3). The prevalence of cancer pain was 66.4% in a recent systematic review (1). In a population-level observational study of patients at the end of life, pain was reported in 69.7% of decedents and 17.2% reported severe pain (2). More than 82.1% of those seeking care from a hospital palliative care unit in Malaysia reported moderate to severe pain upon admission (4).

Morphine is an effective analgaesic recommended for cancer-related pain (5–10). However, for decades, economic aspects, legislation and policy, lack of knowledge and societal attitudes have contributed to inadequate access to medicines for pain relief (11, 12). Negative attitudes and inadequate knowledge among medical practitioners, cancer patients and caregivers also add to the challenges (13). Concerns about opioid addiction and tolerance and their side effects have also been disclosed (4, 14–16). An early study in 2008 suggested that in Malaysia, fear of addiction (36.5%), fear of respiratory depression (53.1%) and poor knowledge of the use of morphine were significant barriers to effective cancer pain treatment (16).

Nevertheless, it has also been reported in a qualitative study that the use of opioids for cancer pain management is acceptable among patients and caregivers in Malaysia, who generally trust doctors’ recommendations and professionalism (4). Therefore, it is essential to improve current cancer pain management by closing the gap in the knowledge, attitudes and perceptions of the use of opioids for cancer pain management among doctors.

To date, the literature on knowledge, attitudes towards and perceptions of cancer pain management is limited in Malaysia. After a decade since the early report was produced in the country on the barriers to cancer pain management (16), this study was designed to assess the knowledge, attitudes and perceptions of morphine use in cancer pain management in doctors. Comparisons in these three aspects were also made between house officers, medical officers and specialists.

## Methods

This cross-sectional study was undertaken at Sultanah Bahiyah Hospital, a major general hospital in northern Malaysia with palliative medicine speciality services. Doctors from multiple disciplines other than palliative medicine who treated cancer patients were invited to participate in the study. They were recruited using the convenience sampling method between 1 November 2020 and 31 December 2020. This study was approved by the Medical Research and Ethics Committee of the Ministry of Health.

After providing consent, the respondents were required to complete a 39-item self-reported questionnaire. The first part of the questionnaire gathered the characteristics of the respondents, while the second part focused on knowledge, attitude towards and perception of cancer pain management. The respondents were instructed to respond to each question on a 5-point Likert scale (1 = strongly disagree; 5 = strongly agree). Only ‘agree’ and ‘strongly agree’ were considered correct or positive responses, except for nine questions worded in the opposite direction.

The study team constructed the questionnaire based on their clinical experience and the existing literature (17–20). Content validity was confirmed by a panel of experts in the field of palliative and pain medicine, who were requested to give each question a score in the range of 1 to 4 according to their relevance. The questions were repeatedly revised until each of them was scored 3 or 4 by all the experts.

The sample size of this study was estimated based on the assumption that 76.1% of the doctors did not have adequate knowledge of cancer pain management (21). The minimal sample size required was 308, with the non-response rate, confidence level and precision set at 10%, 95% and 5%, respectively. The Statistical Package for the Social Sciences (SPSS) software version 26.0 (IBM, New York) was used for the statistical analyses. The findings were summarised as frequencies and percentages. Associations between variables were confirmed using Pearson’s chi-squared and Fisher’s exact tests, with a *P*-value of 0.05 considered statistically significant. Fisher’s exact test was used when the assumption of the chi-squared test was not met (cells with an expected count of less than five were more than 20%).

## Results

### Characteristics of Respondents

Of the 321 respondents, the majority (65.4%) were female (Table 1). Most of them were house officers (64.2%) with less than 2 years of service, followed by medical officers (21.2%) and specialists (14.6%). Only 7.2% had received formal palliative care training before this study. Formal training is defined as hospital or community attachment or structured educational sessions in palliative care. However, most (67.7%) had experience dealing with family members and friends requiring palliative care.

### Knowledge of Cancer Pain Management

Most of the respondents knew the usefulness of the World Health Organization (WHO) analgaesic ladder in morphine use (73.5%) and oral morphine as the first-line therapy for moderate to severe cancer pain (60.7%) (Table 2). Most were also aware of the need to add rescue morphine for breakthrough pain (91.9%) and constipation is a common side effect of morphine (83.1%). However, more than 80% of the respondents believed that intravenously administered morphine is more potent compared to oral morphine and morphine dose escalation is due to tolerance. Meanwhile, less than a quarter of them knew that morphine had no ceiling dose. More than 60% of them were also unaware that pethidine is not recommended for cancer pain management. Specialists demonstrated the best knowledge of cancer pain management, followed by medical and house officers (Table 3).

### Perception and Misconception of Morphine Use

A total of 262 (81.7%, *P* = 0.002) perceived correctly and strongly disagreed or disagreed that patients who used morphine would die sooner (Table 4). Furthermore, 270 (84.1%, *P* < 0.001) had a correct perception by strongly disagreeing or disagreeing that morphine will shorten life expectancy. Respondents strongly agreed and agreed (*n* = 295, 91.9%, *P* < 0.001) that morphine allows the patient to be comfortable in the terminal phase, with 100% agreement in medical officer and specialist positions. The majority correctly perceived morphine use as not a sign of giving up (*n* = 294, 91.5%, *P* = 0.055). The breakdown of the analysis of correct perceptions based on positions is presented in Table 5.

Around one-third of the respondents (34.2%, *P* < 0.001) perceived that morphine use would cause addiction, and one-third were unsure or neutral. Only 37.1% strongly disagreed or agreed that using morphine in cancer pain management in palliative care settings causes addiction. The majority (*n* = 186, 57.9%, *P* < 0.001) strongly agreed and agreed when asked about their worry about respiratory depression in patients taking morphine.

A total of 254 respondents (79.1%, *P* = 0.365) perceived themselves as knowing how to assess pain in their patients, but 45.8% (*n* = 147) of the total respondents were unsure about how to manage the side effects of opioid, and 19.6% did not know how to manage morphine side effects. More than half (51.1%) knew that the patient’s deterioration was not due to the side effects of morphine.

### The Barrier to Morphine Use

Medical officers and specialists ordered morphine prescriptions; therefore, we calculated the degree of agreement on barriers to prescribing only among this cohort (*n* = 115) (Table 6). Though morphine was available, 18.3% (*n* = 21) of respondents answered that they have limited access to morphine. Although there was no dose limitation imposed by hospital administration or pharmacy, interestingly, 27.8% (*n* = 32) strongly agreed and agreed that there was a maximum dose that respondents were allowed to prescribe. The time needed to explain and educate the patient about morphine use was not one of the barriers to prescribing for 63.5% (*n* = 73). Generally, 222 (69.2%) participants strongly agreed and agreed that there were inadequate training opportunities in cancer pain management.

## Discussion

This study provides an overview of the three elements surveyed: knowledge, perception of and barriers to morphine use and managing patients with cancer pain. This survey utilised a self-developed questionnaire focused on doctors managing cancer pain from departments other than palliative medicine and in different positions. Participation in the study was voluntary and most respondents were house officers or interns. This study provided insight and perhaps a reflection on the undergraduate curriculum on cancer pain and palliative medicine exposure.

We examined the questions on knowledge individually to identify areas of improvement for future training. The majority were aware of the WHO analgaesic ladder as a guide for analgaesic use in managing cancer pain and the use of oral morphine as a first choice of analgaesia for moderate to severe cancer pain, which is also reflected in a recent local study in East Malaysia (22). Awareness of the WHO analgaesic ladder has been highlighted as a factor in better knowledge of cancer pain management among young doctors (19). Many knew the importance of maintenance doses of immediate-release morphine for chronic cancer pain management, and more than 90% were aware of the concept of breakthrough pain. This finding is vital for a round-the-clock medication prescription and education for a patient on taking rescue doses. Perhaps the findings reflected our Pain-Free Hospital (PFH) programme.

There were four questions on knowledge that were commonly answered incorrectly. The first was regarding intravenous morphine being more potent than oral morphine and the second was on the assumption that morphine has a ceiling dose. This is still a common knowledge deficit in other regions, especially among younger doctors (19). It is crucial to educate patients on these issues to ensure that adequate opioid analgaesic doses are prescribed for patients with uncontrolled chronic cancer pain. This is reflected in the third question, where the majority still considered morphine dose escalation in cancer treatment to be a result of tolerance. Fear of opioid tolerance should not delay initiating or increasing the opioid dose for cancer patients with pain (23). Fourth, almost half of the respondents (43.3%) doubted whether pethidine was used to manage chronic cancer pain. Only 37% of respondents answered correctly that pethidine was not recommended in such scenarios. Pethidine has been mentioned explicitly in local clinical practice guidelines and should not be used in chronic cancer pain management (23).

There was also a difference in scores regarding knowledge of cancer pain management based on years of service as a doctor. Most of the respondents (79.1%) perceived themselves as knowing how to assess pain, but more than half of the participants did not know or were unsure about how to manage the side effects. Our survey revealed that medical officers (residents) and specialists have better knowledge from more years of training and experience in managing patients with serious illnesses than house officers or interns. This is also reflected in a local study performed in a tertiary hospital to identify doctors’ knowledge and attitudes on pain assessment and management, where less than 50% of healthcare workers with two years of working experiences had adequate knowledge (22). Similarly, several studies overseas have demonstrated that years of service experience correlates with better knowledge of cancer-related pain treatment with morphine (19, 24–27).

Most of the respondents (81.7%) disagreed that morphine would shorten their lives and 91.9% agreed that the use of morphine would allow patients to be comfortable. Though this could reflect a more positive attitude due to continuous education and increased awareness, fear of addiction and respiratory depression still emerged. These fears were like various studies conducted around the globe (13, 17, 18, 20, 28). In 2008 local data, the fear of addiction was 36.5% (16) and after a decade of progress in cancer pain management, the finding did not change much. A total of 34.2% of the respondents expressed similar concerns about addiction. Fear or concern about respiratory depression was still very prominent at 57.9% in our institution, like previous local data by Lim (16) which was 53.1%. The concern about respiratory depression re-emerged again in a local study, as reported by Dharmalingam and Muniandy (22), where only 24.1% answered correctly when the cohort was tested on the likelihood of the patient developing clinically significant respiratory depression in one of the questions. Our findings were like the previous study, which showed a general increase in awareness of morphine use for analgaesia and comfort but were unable to address the fear of addiction and respiratory depression (20). These misconceptions are not exclusive and are commonly seen in other countries. Bertrand et al. (29) reported opiophobia among healthcare providers, especially those with less experience and the major contributors were fear of side effects and addiction.

Although the level of knowledge of healthcare professionals is one of the main factors in adequate morphine use for cancer pain management, perceptions of patients and the public and healthcare system issues with drug accessibility were highlighted as among the critical barriers to cancer pain management in a local publication in 2008 (16). We reviewed only the data among medical officers and specialists on the barriers to prescriptions. This was due to junior doctors or house officers being undertrained and less likely to be encouraged to decide on opioid prescriptions independently. In unpublished local data, Nambbiar and Khoo (30) revealed that 18% of government hospitals did not stock oral morphine solution and 81% of hospitals with oral morphine solution did not have enough to supply a hypothetical patient with a dose of 20 mg every 4 h for a month. All types of opioid preparations available in our country are available at our institution. Although there was no limited access to short-acting oral morphine at our institution, 18.3% of respondents felt they had limited access to morphine. There was also no limitation in prescribing doses at our institution. However, 27.8% agreed that there was a maximum dose they were allowed to prescribe. This was perhaps a mirror of the clinician’s familiarity with and confidence in prescribing morphine for cancer pain management. This was reflected again in the local study, where a dose of 20 mg every 4 h was considered a high dose of morphine (30). In a study by Zin (31) in 2020, Malaysia recorded the lowest total increase in opioid consumption without methadone in 10 years, spanning from 2005 to 2014. The increase is only 47.23% compared to Vietnam, Singapore and Thailand, which recorded an increase of over 100% in 10 years and Indonesia, with the highest data at a 591.67% increment. The country’s overall opioid consumption without methadone is perhaps the most reflective of pain management, particularly for cancer pain, minus misleading data on the use of methadone for harm reduction programmes in Malaysia.

It was reassuring that around two-thirds (63.5%) felt that the time needed to educate the patient on morphine use was not one of the barriers to prescribing. No single barrier was perceived as the main limitation or barrier in morphine use from the patient and family aspects identified from the clinicians’ point of view. It is unknown whether our patients would have other misconceptions about addiction and morphine’s side effects. Studies have reported that healthcare practitioners and the public may have similar negative perceptions towards morphine use in cancer pain control, mainly regarding addiction and side effects (32, 33). In a recent local qualitative study, almost half of the participants from both the caregiver and patient groups still regarded morphine as a substance that can cause dependence, and its use should be a last resort (14). Thus, it is important to learn more about our local community’s perceptions to complement this information further. Respondents were divided when asked if patients were reluctant to report pain. However, this only reflects the clinician’s perception, rather than the genuine opinions of patients and their families.

Malaysia’s PFH programme was introduced to our institution in 2016 and our hospital was awarded the periodic certification of PFH on 3 September 2019 for 3 years. Among the criteria for certification were adherence to several recommended practices to increase better detection and management of pain in all patient settings. The educational materials and methods focused on general pain management in the acute pain setting, with a small segment dedicated to chronic cancer pain management. A series of mandatory clinical practices and administrative interventions were implemented to improve the assessment and management of pain. Among the activities included were regular patient pain score assessment upon interaction with healthcare professionals, continuous education for healthcare providers, including an allied health team, a pain management guide on clinical computer desktops and a communication board in each ward and department. These interventions were initiated to increase the awareness and knowledge of providers in recommending the best care for clients needing pain management, although the focus was mainly on acute pain.

This survey was conducted after 1 year of PFH certification. As the COVID-19 pandemic hit us, healthcare activities and resources focused on managing the infectious disease and curbing its spread to the public. Healthcare professional interinstitutional transfers during the pandemic were also minimal. Though this study did not illustrate the direct correlation between PFH educational activities and participants’ responses, most healthcare professionals who participated would have been exposed to the educational materials beforehand. Therefore, it is essential to develop appropriate strategies to increase awareness of cancer pain management, particularly in using educational opportunities during the PFH programme to incorporate cancer pain management education and address misconceptions and barriers in morphine prescription for cancer pain.

Despite growing attention and increasing awareness of cancer pain management, we continue to face challenges in this area, especially in our knowledge of the use of morphine for cancer pain management. Apart from a section of educational sessions from the PFH programme, most participants in this study had never received formal palliative medicine training or in-depth cancer pain management training. This is in concordance with several studies done worldwide, as healthcare professionals express concern about inadequate training opportunities (19, 20). This issue was also highlighted as one of the barriers our healthcare professionals face, as most respondents (69.2%) agreed that there were inadequate training opportunities for care providers. These findings highlight the need for educational programmes for healthcare workers on managing cancer pain and training modules for medical students and house officers to prepare them for their future careers. A study by Gustafsson and Borglin (34) suggested that educational intervention for nurses can improve their knowledge and attitudes regarding cancer pain; hence, similar training for doctors can bring a similar result. Patient education and the provision of information on opioid use should also be a priority. Interventions from policymakers in increasing access to morphine in healthcare services also play an important role. With easier accessibility of morphine, healthcare providers can gain more experience and confidence in prescribing to patients in need; furthermore, the public can witness the efficacy of morphine in cancer pain control, thus increasing acceptance of it (30, 35).

## Conclusion

This study demonstrated a general inconsistency in knowledge and the presence of negative perceptions among the respondents, especially the house officers and medical officers. We also identified a few common barriers to morphine usage for cancer pain patients, including perceived limitations in prescribing and familiarity with doses. This will further support the implementation of a more thorough and continuous education programme to improve knowledge among doctors. With good knowledge, there will be correct perceptions, and better care can be provided to cancer patients.

### Limitations

This study has a few limitations. First, this study only represents doctors’ knowledge, perceptions and barriers in a single centre. Furthermore, most respondents were junior doctors still under supervision, with limitations in prescribing in clinical practice. This cohort was not truly representative of the general practice of doctors in our institution. Most prescribing decisions, including opioids in tertiary hospitals, are made by senior doctors, particularly medical officers and specialists. These findings also did not reflect Malaysia’s cancer pain management practice due to variations in resources and the availability of clinical services.

Second, many of our questions were subjective and no clinical scenario questions were used to test the actual knowledge and ability to manage cases. The questions were not comprehensive, which prevented further in-depth analysis. Despite these challenges, most of the data were still consistent with many similar studies, both locally and abroad. Nevertheless, the findings from this study can help provide references to improve knowledge with the perception of cancer pain management and address misconceptions about morphine use for future educational planning.

## Figures and Tables

**Table 1:** Characteristics of respondents (*N* = 321)

Characteristics	*n* (%)
Gender	
Female	210 (65.4)
Male	111 (34.6)
Designation	
House officer	206 (64.2)
Medical officer	68 (21.2)
Specialist	47 (14.6)
Department	
Internal Medicine	101 (31.5)
Obstetrics and Gynaecology	76 (23.7)
Orthopedic	47 (14.6)
Anaesthesiology	28 (8.7)
Surgery	22 (6.9)
Paediatrics	13 (4.0)
Others	34 (10.6)
Duration of services	
< 2 years	212 (66.0)
2–5 years	41 (12.8)
> 5 years	68 (21.2)
Received formal training in palliative care	23 (7.2)
Having experience in dealing with family members and friends requiring palliative care	135 (67.7)

**Table 2:** Knowledge of cancer pain management (*N* = 321)

Aspects	Correct answer *n* (%)	Neutral *n* (%)
The WHO Pain Ladder is used to guide morphine use in cancer patients (A)	236 (73.5)	51 (15.9)
Oral morphine is the first choice of analgesic therapy in moderate to severe cancer pain (A)	195 (60.8)	53 (16.5)
Maintenance doses of immediate release morphine, such as morphine syrup, should be prescribed	241 (75.1)	47 (14.6)
Rescue analgaesia, such as additional morphine, needs to be available for breakthrough pain (A)	295 (91.9)	23 (7.2)
There is a ceiling dose for morphine (D)	75 (23.3)	93 (29.0)
Intravenous morphine is more potent than oral morphine (D)	51 (15.8)	74 (23.1)
The correct dose of opioid is the dose that relieves the patient pain to an acceptable level (A)	234 (72.8)	64 (19.9)
Morphine dose escalation in cancer patients is due to tolerance (D)	60 (18.6)	68 (21.2)
Opioid-induced constipation is a common side effect (A)	267 (83.1)	48 (15.0)
Drowsiness after morphine initiation improves after several days (A)	157 (48.9)	130 (40.5)
It is important to identify causes of cancer pain requiring specific treatment, such as radiotherapy (A)	285 (88.7)	33 (10.3)
Tramadol is an opioid (A)	265 (82.5)	28 (8.7)
Pethidine is recommended for chronic cancer pain patients (D)	119 (37.0)	(43.3)

Notes: (A) Correct answer - ‘Strongly agree’ or ’Agree’; (D) Correct answer - ‘Strongly disagree’ or ’Disagree’

**Table 3:** Correct knowledge about morphine use in cancer pain management by position of the respondents (*N* = 321)

Question	Position, *n* (%)			*P*-value^a^

	House officer	Medical officer	Specialist/Consultant	
The WHO analgaesic ladder is used to guide morphine use in cancer patients	145 (70.4)	48 (70.6)	43 (91.5)	0.010
Oral morphine is the first choice of analgesic therapy in moderate to severe cancer pain	111 (53.9)	42 (61.8)	42 (89.4)	< 0.001
Maintenance doses of immediate release morphine, such as morphine syrup, should be prescribed	141 (68.4)	58 (85.3)	42 (89.4)	0.001
Rescue analgaesia, such as additional morphine, needs to be available for breakthrough pain	182 (88.3)	66 (97.1)	47 (100.0)	0.007
There is a ceiling dose for morphine	13 (6.3)	35 (51.5)	27 (57.4)	< 0.001
Intravenous morphine is more potent than oral morphine	13 (6.3)	22 (32.4)	16 (34.0)	< 0.001
The correct dose of opioid is the dose which relieves the patient pain to an acceptable level	138 (67.0)	53 (77.9)	43 (91.5)	0.002
Morphine dose escalation in cancer patients is due to tolerance	16 (7.8)	21 (30.9)	23 (48.9)	< 0.001
Opioid-induced constipation is a common side effect	154 (74.8)	66 (97.1)	47 (100.0)	< 0.001
Drowsiness after morphine initiation improves after several days	97 (47.1)	33 (48.5)	27 (57.4)	0.439
It is important to identify causes of cancer pain requiring specific treatment, such as radiotherapy	173 (84.0)	65 (95.6)	47 (100.0)	0.001
Tramadol is an opioid	160 (77.7)	62 (91.2)	43 (91.5)	0.009
Pethidine is recommended for chronic cancer pain patients	46 (22.3)	36 (52.9)	37 (78.7)	< 0.001

Note:

a Pearson’s chi-squared test

**Table 4:** Perception on morphine use in cancer pain management (*N* = 321)

Aspects	Degree of agreement		

	Strongly disagree/Disagree *n* (%)	Neutral *n* (%)	Strongly agree/Agree *n* (%)
Patients who use morphine will die sooner	262 (81.6)	46 (14.3)	13 (4.0)
The patient should only be prescribed morphine when the pain is severe	144 (44.8)	59 (18.4)	118 (36.7)
Enduring pain is better for cancer patients	227 (70.7)	50 (15.6)	44 (13.7)
Using morphine is a sign of giving up on cancer patients	294 (91.5)	16 (5.0)	11 (3.4)
Morphine will shorten patient life expectancy	270 (84.1)	41 (12.8)	10 (3.1)
Morphine usage causes addiction	119 (37.1)	92 (28.7)	110 (34.3)
I know how to manage the side effects of morphine	63 (19.6)	147 (45.8)	111 (34.6)
I know when my patients’ deterioration is not due to side effects of morphine	46 (14.3)	111 (34.6)	164 (51.1)
I know how to assess pain in my patients	6 (1.9)	61 (19.0)	254 (79.1)
I am worried about respiratory depression in patients taking morphine	51 (15.9)	84 (26.2)	186 (58.0)
I am able to explain to patients why morphine is required for cancer pain	34 (10.6)	107 (33.3)	180 (56.1)
Morphine allows a patient to be comfortable in the terminal phase	4 (1.2)	22 (6.9)	295 (91.9)

**Table 5:** Correct perceptions of morphine use in cancer pain management by the position of the respondent (*N* = 321)

Aspects	Position, *n* (%)			

	House officer	Medical officer	Specialist/Consultant	*P*-value
Patients who use morphine will die sooner	157 (76.2)	60 (88.2)	45 (95.7)	0.002
The patient should only be prescribed morphine when the pain is severe	58 (28.2)	46 (67.6)	40 (85.1)	< 0.001
Enduring pain is better for cancer patients	131 (63.6)	58 (85.3)	38 (80.9)	0.001
Using morphine is a sign of giving up on cancer patients	184 (89.3)	63 (92.6)	47 (100.0)	0.055
Morphine will shorten patient life expectancy	160 (77.7)	64 (94.1)	46 (97.9)	< 0.001
Morphine usage causes addiction	46 (22.3)	35 (51.5)	38 (80.9)	< 0.001
I know how to manage the side effects of morphine	38 (18.4)	38 (55.9)	35 (74.5)	< 0.001
I know when my patients’ deterioration is not due to side effects of morphine	71 (34.5)	51 (75.0)	42 (89.4)	< 0.001
I know how to assess pain on my patients	160 (77.7)	58 (85.3)	36 (76.6)	0.365
I am worried about respiratory depression in patients taking morphine	12 (5.8)	15 (22.1)	24 (51.1)	< 0.001
I am able to explain to patients why morphine is required for cancer pain	93 (45.1)	48 (70.6)	39 (83.0)	< 0.001
Morphine allows a patient to be comfortable in the terminal phase	180 (87.4)	68 (100.0)	47 (100.0)	< 0.001

**Total**	206 (100.0)	68 (100.0)	47 (100.0)	**–**

Notes:

a Pearson’s chi-squared test

**Table 6:** Barriers to morphine use in cancer pain management among medical officers and consultants (*N* = 115)

Aspects	Degree of agreement, *n* (%)		

	Strongly disagree/Disagree	Neutral	Strongly agree/Agree
I have limited access to morphine	70 (60.9)	24 (20.9)	21 (18.3)
There is a maximum dose for morphine that I am allowed to prescribe	47 (40.8)	36 (31.3)	32 (27.8)
The need to explain morphine and its side effects limits me from prescribing morphine	73 (63.5)	15 (13.0)	27 (23.5)
My medical and nursing colleagues have concerns when I prescribe morphine	57 (49.6)	29 (25.2)	29 (25.2)
